# Spinal Tumour en Bloc Surgery: A Series of Abandoned Surgical Cases

**DOI:** 10.7759/cureus.27758

**Published:** 2022-08-07

**Authors:** Thomas H Land, Yasir A Chowdhury, Yan Ting Woo, Mutasim F Chowdhury, Melvin Grainger, Marcin Czyz

**Affiliations:** 1 Neurosurgery and Spinal Surgery, Queen Elizabeth Hospital Birmingham, Birmingham, GBR

**Keywords:** spinal surgery complication, abandoned, en bloc surgery, total en bloc spondylectomy, spinal oncology, spinal tumour

## Abstract

Select spinal tumors can be treated with en bloc spondylectomy (EBS) but the surgical complexity and relatively low frequency of eligible tumors render EBS an uncommon procedure. The expanded surgical access encompasses acceptance of relatively high morbidity as a trade-off against improved oncological results and survival. EBS durations can be long with dynamic changes affecting the risk-benefit ratio as the surgery proceeds.

We present a series of cases where we have elected to “abandon” EBS due to adverse findings or rising intraoperative risk along with our lessons learned.

A search of our surgical database for all “en bloc” spinal tumor procedures over a three-year period was performed and 27 operations were identified. Of these, four were abandoned. Two of the four surgeries were halted owing to adverse anatomical findings. One involved significant tumor growth from the interval imaging bringing into question disease control and the other displayed tumor adherence to the lung requiring significant dissection. The further two cases incurred significant blood loss and associated physiological complications of end-organ dysfunction.

Pre-operative embolization (POE), anesthetic monitoring, controlled hypotension, volume replacement, and transfusion optimize our chance of achieving the surgical plan. However, cardiovascular instability must be managed promptly and early warning signs of end-organ injury (lactate, renal output) should not be overlooked. In some situations abandoning the procedure may be in the best interests of the patient.

## Introduction

Although en bloc spondylectomy (EBS) is a recognized treatment for certain spinal tumors including primary malignant bone tumors, aggressive benign tumors, and occasionally solitary metastatic tumors, the surgical complexity and relatively low frequency of tumors eligible, prevent this from being a routine technique. En-bloc surgery requires the removal of the entirety of a tumor without violation of its capsule. This concept is familiar to oncological surgery and has been widely accepted to reduce local tumor recurrence and lengthen survival [1-4]. 

EBS, initially described by Stener [5], Roy-Camille [6] and further developed by Tomita [7,8], poses several anatomical and physiological challenges. In comparison to “intralesional” debulking “wide” and “marginal” (dissecting out of the pseudocapsule) [9] gross total excision requires expanded surgical access to dissect the specimen, and physically deliver it out through the wound around preserved neural structures. Adjacent vascular, visceral, and neurological structures generate unique issues at regional spinal levels and circumferential access to the specimen may necessitate combined posterior and anterior approaches. Thoracic and abdominal access, mobilizing great vessels, and greater tissue dissection contribute to increased blood loss, operative time, morbidity, complication rates, and recovery time [10-12]. 

The planning of an en bloc excision may indeed involve the necessary sacrifice of adjacent structures, e.g division of nerve roots, and encompasses acceptance of a degree of morbidity as a trade-off against improved oncological result and survival. We find that going into surgery with this prior calculation can motivate us as surgeons to complete the planned task. We must however be wary of situational changes, not uncommon in long complex surgical procedures, which can alter the risk-benefit balance making our original surgical aims no longer justified. There is a relative paucity of data describing intraoperative decision-making across all surgical specialties, with rare surgical procedures such as EBS being no exception. 

The aim of this paper is to present a case series where the decision was made to “abandon” or halt spinal tumor EBS along with the associated learning points.

## Case presentation

This case series was approved by an internal board review. The surgical database was searched for all spinal tumor procedures labeled as “en-bloc”. Between January 2019 and April 2022 27 EBS cases were identified. A case notes review was performed and of these 27 operations four (15%) were “abandoned”. Our definition of abandoned in this instance was the unplanned halting of the surgical procedure, once commenced, secondary to adverse intraoperative events or findings. 

Operative technique

As there is considerable variation in the surgical technique needed for EBS depending on the spinal level and tumor morphology we cannot report a standard surgical approach and technique for this case series. Surgical planning was made following a multidisciplinary team (MDT) discussion involving radiologists, surgeons, clinical and radiation oncologists as well as histopathologists for all patients. Anterior, posterior, or combined surgical approaches were used as appropriate following the principles of blunt anterior vertebral body dissection, ligation of radicular arteries, circumferential decompression of the spinal cord, and atraumatic handling of the spinal cord as described by Tomita et al. [8]. Vascular tumors were treated with pre-operative embolization (POE). Primary rigid reconstruction varied with each case using an expandable cage or autologous bone graft for anterior stabilization along with posterior instrumentation. Reconstruction techniques have been well documented previously [13,14] and the discussion of these in-depth is beyond the scope of this study. Osteotomies were completed with Sonapet® (Stryker) and osteotomes. In all cases involving major manipulations or resection of the vital anatomical structures adjacent to the tumor or spine, direct input from specialists from the relevant field was obtained.

Anesthetic considerations

All patients were seen in the pre-operative assessment clinic by a consultant anesthetist for review and optimization of medical co-morbidity. Intensive care unit (ITU) beds were booked for the post-operative period as standard. Routine pre-op bloods included the cross-matching of four units of blood. Central line access and arterial line monitoring were employed, along with double lumen endotracheal (ET) intubation if deflation of a lung was required. Intraoperative cell salvage was used for as long as possible depending on the requirement for thrombin-based hemostatic agents. We used relative hypotension to control blood loss during surgery (80-100mmHg systolic pressures). Along with typical anesthetic monitoring, particular attention was paid to urine output and serum lactate measurements to monitor end-organ function. Blood gas Haemoglobin (Hb) levels and thromboelastography (TEG) were used for real-time hemorrhage and clotting assessment. 

Case one: 27-year-old male biopsy-proven giant cell tumor (GCT)

**Figure 1:**
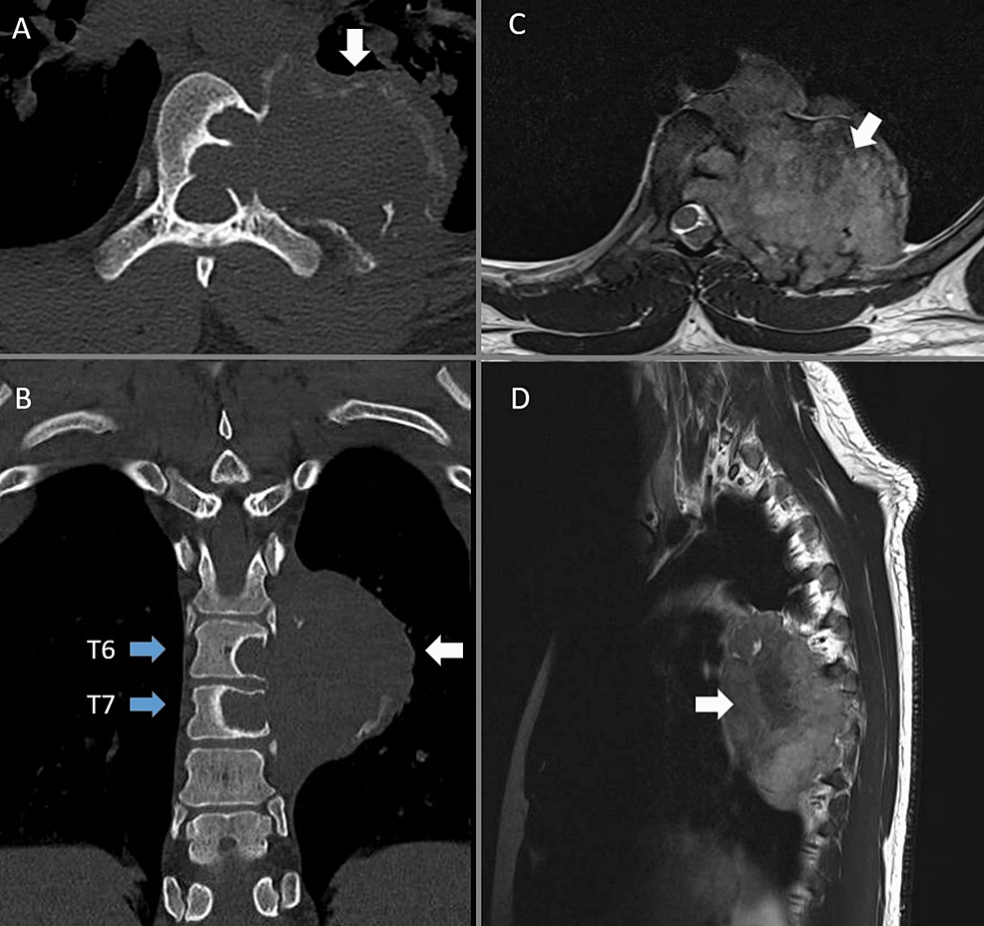
Case One Twenty-seven-year-old male with a giant cell tumor. A and B demonstrate axial and coronal CT views with lytic tumor evident within T6 and T7 (blue arrows), the extraosseous extend from T5-T8 can be seen in A and B (white arrows). C and D show axial and sagittal T2 MRI views with evident tumor mass (arrows).

Case one presented with several months of left-sided chest well pain. Subsequent imaging showed a lytic tumor at T6/7 growing against the left lung (6.3 x 6.5 x 6.4 cm). He was initially treated with Denosumab therapy within one month of diagnosis. After initial growth (to 8.8 x 8.5 x 9.7 cm) the tumor became stable but growth commenced again after one year. At this time T6 and T7 were involved with extraspinal extension from T5-T8 (Figure 1). Following new left leg pain, imaging demonstrating compression of the spinal cord prompted a surgical intervention. POE of the feeding T5-7 intercostal vessels was performed the day before surgery. 

A two-staged operation (posterior-anterior approach) was planned as a single sitting. Stage one included an instrumented fusion of T3-T10, T5, and T8 laminectomy followed by T6 and T7 left hemilaminectomy, costotransversectomy, and pediculectomy. Right-sided paravertebral dissection was performed down to the aorta. On the left side, the lung was strongly adherent to the tumor. Osteotomies and discotomies were made to release the left lateral aspect of T5 and T8 vertebral bodies along with the complete T6 and T7 vertebrae.

The planned stage two thoracotomy access to the left chest was postponed owing to the adherent lung, long operative time (8 hours), and developing deranged acid-base balance (Lac 2.67, BE -5.0).

On waking the patient was found to have anterior cord syndrome with flaccid paralysis of the lower limbs thought to be secondary to hypoperfusion.

A left-sided thoracotomy was performed after three days, with cardiothoracic surgeon assistance concluding that lobectomy would be required to facilitate EBS. Significant desaturation (35%) and decreased cardiac output occurred on deflating the left lung. Repeated repositioning of the double-lumen ET tube failed to improve the parameters. The decision was made to abandon surgery and return to ITU. Consolidation was seen on the chest x-ray and treatment for hospital-acquired pneumonia was commenced. At two weeks we considered converting our plan to intralesional debulking but he was able to tolerate single lung ventilation and the lobectomy was completed with the delivery of specimen en bloc. Anterior reconstruction was performed with a cage and autologous rib graft. He made a good respiratory recovery and improved neurologically, being able to mobilize with the aid of two crutches.

Case two: 63-year-old female with T5 thyroid metastasis

**Figure 2:**
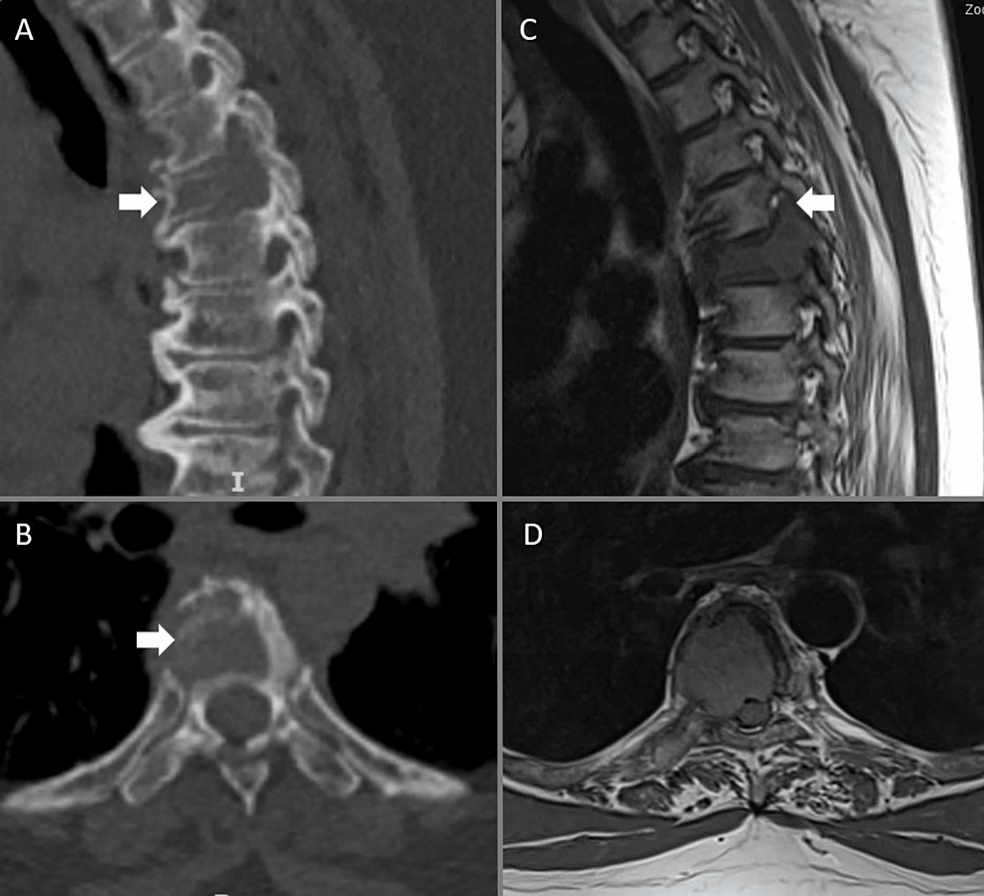
Case two Sixty-three-year-old female with thyroid metastasis. A and B demonstrate a lytic T5 lesion on sagittal and axial CT imaging respectively (arrows). C and D show right parasagittal and axial T1 MRI images. Of note in C tumor can be seen extending along the T5 right pedicle into the superior articular process (arrow).

Case two, an asthmatic and long-standing type two diabetic presented with 18 months of thoracic pain radiating to the right chest wall, burning thigh pain, and proximal lower limb weakness. Her background included right hemithyroidectomy 20 years ago for benign disease, three years of left thyroid swelling, and completed thyroidectomy six months previously. Staging demonstrated PT3 papillary thyroid carcinoma with T5 (Figure 2) and right iliac metastases.

Following the MDT discussion with consideration of the past medical history and all of the treatment options, EBS of the T5 tumor was planned. POE was performed and the predominant supply from the right T5 segmental artery embolised. A small right T4 feeder could not be navigated and was left. Owing to difficulty securing an ITU bed EBS was performed five weeks later.

EBS was planned as a posterior approach. Initially, instrumentation of T2-T8 was performed followed by left fifth/sixth rib osteotomy and transverse process removal. Subsequently, retro-pleural dissection and then division of the left T5 nerve root and segmental vessels at aortic origin was completed. On the right the 5th and 6th ribs were osteotomised, the pleural cavity opened (preserving pleura over specimen as an extended margin) and the right T5 nerve root ligated along with small segmental vessels. Left hemilaminectomy of T4, T5, and left facetectomy of T4/5, T5/6 were completed. The right T4 pars were divided to leave the right T4/5 facet (with tumor involvement) intact. Osteotomies just above the T4/5 disc and below the T5/6 disc were commenced, with just the anterior-most bone remaining, when a significant drop in blood pressure (BP) and tachycardia occurred. Her Hb was stable and BP improved with fluids, however on the basis of rising lactate (lactate 2.45, BE -5.9, pH 7.31) and relative anuria for the last hour we felt the completion of osteotomies could risk further bleeding and left her vulnerable to further deterioration. Hemostasis was achieved and the wound closed. 3000ml crystalloid, three units packed red cells and 500ml cell salvage were given and the patient was moved to ITU sedated.

Overnight, acidosis worsened and the following day the decision was made to wake up rather than return to surgery. Episodes of hypotension, desaturation, and supraventricular tachycardia were treated in ITU over the next four days prior to ward discharge. Return to the theatre was conducted at one week for posterior wound reopening, completion of osteotomies, and anterior soft tissue divisions. Unfortunately, fracture of the very friable tumor resulted in removal as three segments rather than en bloc. Reconstruction was completed with an expandable cage and rods. Our patient remained in ITU for two days, had a chest drain for five days, and was discharged after 10 days of mobilizing with a frame. 

Case three: 61-year-old female with malignant peripheral nerve sheath tumor (MPNST)

**Figure 3:**
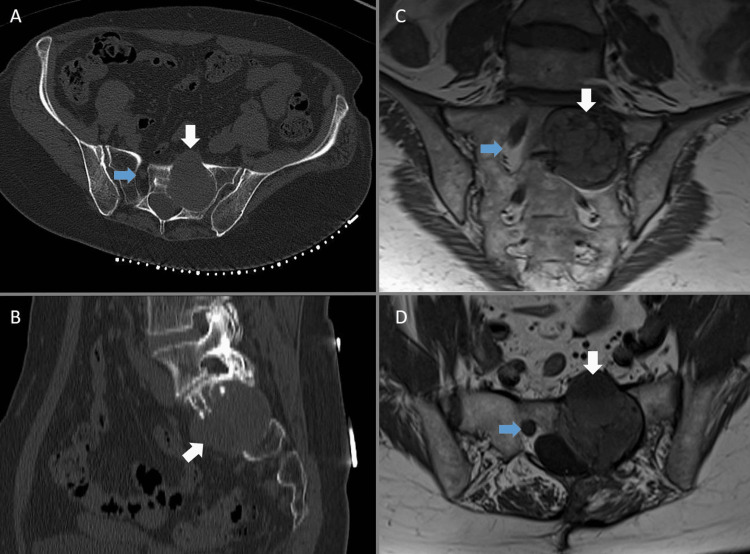
Case three Sixty-one-year-old female with malignant peripheral nerve sheath tumor originating from the left S1 nerve root. Axial and sagittal CT views (A and B) demonstrate a lytic left sacral mass centered on the left S1 foramen. Further imaging with coronal and axial T1 MRI is included in images C and D. The tumor is marked by the white arrows. The normal right S1 foramen for comparison is marked by the blue arrows.

Case three had a previous history of left S1 MPNST with intralesional debulking and biopsy performed at another institution. Imaging demonstrated a heterogenous solid/cystic enhancing left sacral mass of 4 x 4.8 x 5.2cm (Figure 3). Given the diagnosis and previous intralesional procedure, the chance of a cure was limited. En-bloc resection was, however, felt technically feasible and the most likely option to provide optimal local control of the disease. A two-stage, same-day, combined anterior-posterior approach hemisacrectomy as a joint procedure with retroperitoneal sarcoma general surgeons was planned. Initially, cystoscopy and bilateral prophylactic ureteric stents were inserted followed by laparotomy (stage one). The sigmoid colon left ureter and iliac vessels were identified and mobilized to access the left sacroiliac joint (SI). Then the sacral and obturator veins were ligated to access the ischiatic notch. A planned ligation of left S1 and S2 nerve roots, vertical osteotomy from L5 disc to S2 foramen, the section of L5 disc, and horizontal osteotomy from S2 foramen to ischiatic notch were completed prior to closure. Stage one was completed in three hours with 1500ml blood loss.

The second stage was commenced prone with muscle strip for exposure of the sacrum, left alar, and SI joint superiorly and left sciatic notch inferiorly. L5 and S1 laminectomy was completed and posterior dura opened. The left S1 and S2 nerve roots were identified and divided intradurally before the anterior dura was opened. At this stage, ongoing significant bleeding was noted. Following a hemostatic pause to evaluate, the developing scenario was hemodynamic instability and anuria, likely secondary to blood loss, deranged clotting, and renal hypoperfusion (lactate 2.10, BE -0.4). Massive blood loss transfusion protocol was followed but a unanimous decision to defer further osteotomies to another time was made. Hemostasis with hemostatic products was achieved and the wound closed. We estimated 5000 ml of blood loss in total. The patient went to ITU for correction of TEG-based clotting with cryoprecipitate and platelets, Hb correction with packed red cells, and hemofiltration. After five days in ITU renal function improved but low platelet count and albumin led the focus to change from when to return, to whether to return for the completion of EBS. A decision was made not to proceed. She did return to the theatre after six days for laparotomy, washout, and removal of swabs left intentionally for hemostasis, washout of the sacral wound, and further dural repair. Her 24-day ITU stay was further complicated by flash pulmonary edema, anemia, renal failure, S. epidermidis bacteremia and extended-spectrum beta-lactamase (ESBL) wound infection. On discharge three weeks later she was mobile around the room independently and using a handrail for stairs. Further elected treatment was with radiotherapy. 

Case four: 26-year-old female with T8 Osteosarcoma

Case four had a background of adolescent idiopathic scoliosis and posterior stabilization aged 16. Subsequently, she presented one year ago with acute paraplegia of grade B on the American Spinal Cord Injury Association (ASIA) impairment scale and was diagnosed with T8 osteosarcoma and lung metastases. She was treated with emergency posterior decompression and post-operative chemotherapy at another unit. Thoracoscopic resection of pulmonary metastases demonstrated histological good response with 100% necrosis following chemotherapy. Following the MDT discussion, given this good response, the initial consideration was radical resection with the sacrifice of the cord. However, upon clinical review, she had preservation of protective sensation in the legs and a recent recovery of movement in her toes. A cord-saving en bloc excision, to aim to preserve her residual function and provide optimal local control of her spinal disease, was elected.

**Figure 4:**
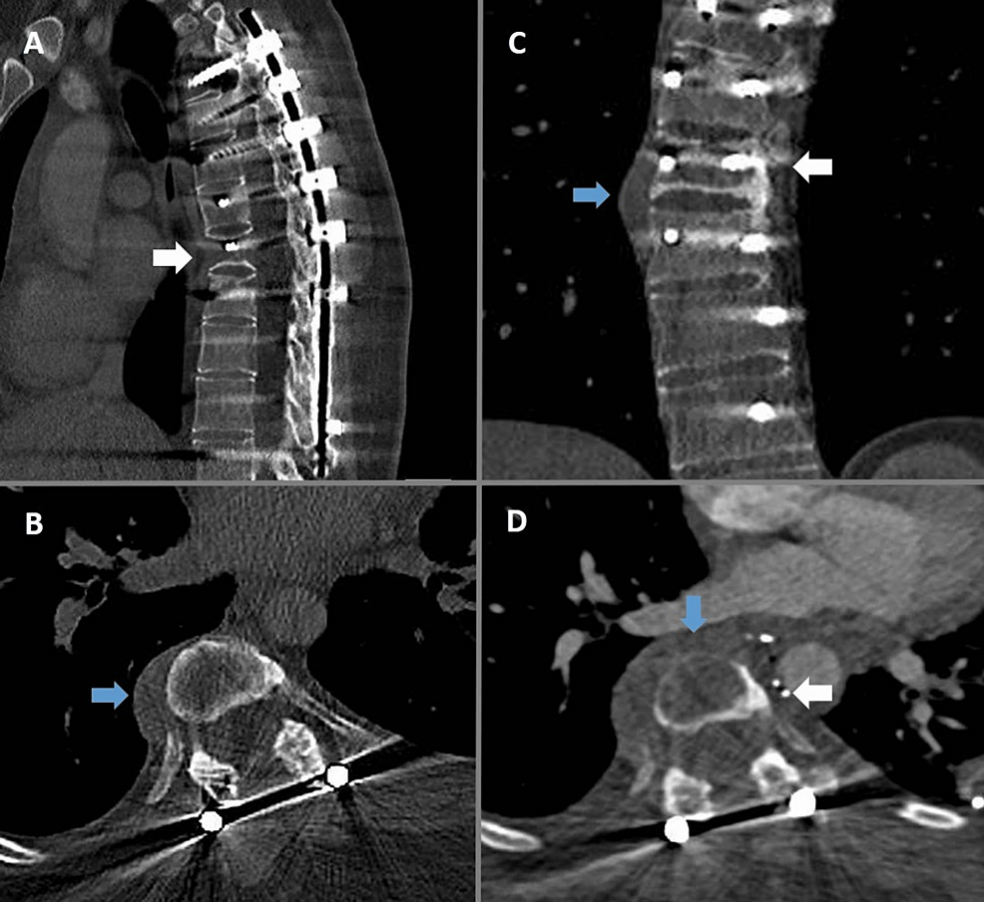
Case four Twenty-six-year-old female with T8 Osteosarcoma. All images are plain CT scans (A-C show pre-op imaging). Image A is a sagittal view demonstrating the former construct. Image B demonstrates an axial view through T8. Image C is a coronal view. Extraosseous tumor mass is indicated by the blue arrows and T8 Level by the white arrows. Image D is a post-thoracotomy axial image demonstrating an increased tumor anterior to T8 (blue arrow). Some surgical clips can be seen between T8 and the aorta (white arrow).

Her MRI four weeks prior to surgery demonstrated signal change within T8 and superior T9 likely to represent residual tumor, with a small right paraspinal extraosseous component. Owing to the artifact around the metalwork on MRI images we present CT imaging (Figure 4). Combined anterior-posterior surgery with stage one thoracotomy, for anterior dissection/release, and stage two removal of posterior metalwork with posterior osteotomies for T8 spondylectomy was planned.

A left-sided thoracotomy with fifth rib osteotomy (retaining vascular pedicle for use as strut graft), was performed followed by left lung deflation, the opening of pleura, and mobilization of the aorta. The intraoperative appearance was of significant extraosseous growth T7-T9, including the plane between the aorta and esophagus. The degree of progression from the scan one month previously suggested aggressive tumor behavior. Frozen section histology taken from a small tumor breach showed a high-grade pleomorphic tumor. A discussion was held with oncology regarding tumor progression, reservation about prognosis, and the validity of performing the second stage as operative recovery time would be significant with limited oncological benefit. A decision was made to abort the procedure. The thoracotomy was closed with a chest drain. CT staging was repeated showing an increase in the size of a right lower lobe pulmonary metastasis hence the disease was not controlled as previously thought. She was discharged from ITU after five days and went home after a further two days with the ability to transfer and mobilize in a wheelchair as per pre-op. Follow-up was planned with further chemotherapy. 

## Discussion

Case selection and planning

Careful patient selection is essential prior to EBS. Given that the aim of EBS is often for local disease control rather than curative purposes the systemic disease burden has a great effect on long-term outcomes. In case four we found unfavorable intraoperative disease with progression from our imaging four weeks prior. We should be wary when operative justification rests on systemic staging suggesting disease remission. Our current routine is to perform an up-to-date whole spine MRI scan 24-48 hours prior to the operation which minimizes the risk of omitting the asymptomatic tumor growth during the time between the clinic review and surgery. In rare cases, it can also reveal further spinal lesions which would have a major impact on surgical planning.

In addition to the spinal surgery and anesthetic team complicated combined approaches often require the presence of specialist assistance (e.g. second spinal consultant, cardiothoracic, general surgical, plastics, or sarcoma surgeon). Relevant specialist and oncologist opinions may be sought as appropriate.

Blood loss

Two of our four cases suffered deterioration from effects of hemorrhage and hypotension. On both occasions, the deterioration coincided with making partial osteotomies, a generally accepted crux point in bleeding for EBS. 

One of the major concerns with EBS is the accepted high volume hemorrhage and associated risks. Most of the adverse intraoperative events are either directly or indirectly related to blood loss. Most of our management strategies are aimed at minimizing, and compensating for, hemorrhages. Vertebrae have copious blood supply potentially increased further with adjacent tumor involvement. Non-compressible vascular channels in the cancellous bone can make bleeding difficult to control. Given the aim of EBS is not to breach the tumor capsule there is some advantage gained by reducing blood loss from vascular tumor types that would be readily encountered with intralesional surgery. Generally, however, this advantage is offset by bleeding from increased complexity, dissection, and operative length. Spinal tumor surgery variables influencing blood loss include histology, epidural spinal cord compression, tumor location, and body mass index (BMI), as also surgical strategy and procedure length [15]. Hypervascular tumors, en-bloc surgery, and the presence of extraosseous tumor mass have been shown to be independent variables for blood loss [16]. Estimated blood loss for EBS from systematic reviews varies from 1742ml to 4867ml [1,17]. Transfusion rates for combined anterior-posterior thoracic oncological procedures are reported from 17-70% [18]. 

The vascularity of tumors varies [19] and although the aim of EBS is to remain outside the tumor capsule we generally accept that for the more vascular tumors POE improves safety. POE aims to reduce blood loss [20] and operative time, increase the potential resection possible and improve field visibility. Following POE in spinal tumor surgery evidence suggests an average blood loss of 1600ml [21] although much of the literature does not define EBS in subcategory analysis. Embolization is sometimes contraindicated given shared radiculomedullary cord supply. Generally in our unit, we aim to embolise metastatic tumors and giant cell tumors pre-operatively. Case two received embolisation of her thyroid metastasis five weeks prior to EBS, which could have provided a window for significant revascularisation leading to intraoperative bleeding and hypotension. It is generally recommended that POE is performed as close as possible to surgery. However, some evidence suggests there is no significant difference in surgical blood loss following POE for immediate vs delayed (24-48hr) surgery, although longer delays are not evaluated [22].

We normally choose to run relative hypotension of 80-100mmHg systolic pressure as a balance between reducing bleeding and maintaining adequate tissue perfusion to minimize end-organ injury. Faced with hemorrhage at our unit we are guided by volume replacement in the first instance to maintain cardiovascular stability, whilst monitoring Hb on blood gas samples to guide the use of allogenic transfusion or cell salvage replacement (target Hb > 80g/L). A further complication of coagulopathy can occur with red cell replacement and when larger transfusion protocols are initiated (> 4 units) often platelets and clotting factors need to be considered. TEG can be very useful to determine real-time clotting status. We also consider the use of IV tranexamic acid, which has been shown to reduce gross blood loss [23] and transfusion [24] in spinal surgery. 

Anesthetic time

It is widely accepted that certain complication rates increase with longer operative times. Infections for example increase significantly after two hours duration. Cheng et al. [25] find that complications increase by 14% for every additional 30 mins of surgical time. However, evidence is generally based on shorter surgical times and there is a paucity of data for long operations. The average operative length for EBS varies from 390 min [17] to 1195 min [1] (two-stage combined approach). It is logical that blood loss can increase with longer operative times but other factors also come into play such as tissue hypoperfusion, oxygenation, and end-organ injury which although highly dependent on dynamic cardiovascular stability are likely to be cumulative effects. Surgeon fatigue should also not be forgotten. We feel that the absolute length of surgery is not as crucial as the events occurring within the surgery. Features of end-organ dysfunction such as rising lactate or anuria can be linked to long-term adverse outcomes [26,27]. Lactate level is affected by tissue oxygen deficit, ventilation, oxygen-carrying capacity, and cardiac output. Rising lactate should be interpreted as an important warning sign. Three of our four abandoned cases demonstrated rising lactate in the background of operative difficulties with hemorrhage control or ventilation. Two of these cases developed clinically significant end-organ injury (one with renal failure requiring hemofiltration, one with spinal cord injury). 

Staging surgery

Our current elective planning for EBS follows MDT discussion and identification of appropriate cases. However, we do not currently have a formal assessment system for assessing risk and operative time. Factors we take into account are tumour type, spinal location, approach (posterior vs anterior vs combined), comorbidity and number of vertebrae affected. Mirza et al score spinal surgery “invasiveness” determining the effect of surgical extent on blood loss and operative time [28], however, this does not account for specifics of EBS. Several staging systems are used in spinal oncology for prognosis and surgical decision-making. Tomita et al. [7,8] have a surgical classification of spinal tumors but there is no literature linking this to complication rates or operative time. Using the above factors we estimate whether the surgical plan can be achieved in a full day theatre session (10 hours) with or without extended hours overrun. Anesthetic time and setup as per our described operative technique must be included. 

Logic dictates that a single-stage procedure be performed in one sitting where staging based on a need for repositioning in combined approach cases may indicate a natural break. However one significant barrier to performing an elective two stage plan on separate days is the logistical difficulty faced by obtaining theatre space, in conjunction with ITU capacity, on two days relatively adjacent in time. Another issue with a planned surgical gap is the concept of anaesthetic window. The majority of post-op complications occur on days one-three and the sequelae may persist for longer [29,30] rendering a patient unfit. Hence the safest strategy for the patient may be returning to the theatre the next sequential day otherwise optimization may be unpredictable.

## Conclusions

Optimal setup and flexible approach to the surgical plan based on the intraoperative findings and feedback do play a major role in achieving favorable outcomes in spinal en bloc procedures. Careful physiological monitoring allows the identification of situations in which abandoning the resection works in the best interest of the patient. Sometimes we must abandon our best-laid plans but in certain cases, it is possible to recommence the procedure once the deranged patient’s physiology has been optimized.
